# The autophagic regulation of rosiglitazone-promoted adipocyte browning

**DOI:** 10.3389/fphar.2024.1412520

**Published:** 2024-06-04

**Authors:** Yue Li, Wanqing Zheng, Xinhang Li, Zhengwei Lue, Yun Liu, Jiaying Wu, Xiangnan Zhang

**Affiliations:** ^1^ National Key Laboratory of Advanced Drug Delivery and Release Systems, College of Pharmaceutical Sciences, Institute of Pharmacology and Toxicology, Zhejiang University, Hangzhou, China; ^2^ Jinhua Institute of Zhejiang University, Jinhua, China; ^3^ Zhejiang Provincial Key Laboratory for Drug Evaluation and Clinical Research, Department of Clinical Pharmacy, The First Affliated Hospital, Zhejiang University School of Medicine, Hangzhou, China

**Keywords:** rosiglitazone, PPARγ agonist, autophagy, mitophagy, adipocyte browning, obesity

## Abstract

**Objective:** Browning of white adipocytes is considered an efficient approach to combat obesity. Rosiglitazone induces the thermogenetic program of white adipocytes, but the underlying mechanisms remain elusive.

**Methods:** Expression levels of browning and autophagy flux markers were detected by real-time PCR and immunoblotting. H&E and Oil Red O staining were performed to evaluate the lipid droplets area. Nuclear protein extraction and immunoprecipitation were used to detect the proteins interaction.

**Results:** In this study, we reported that rosiglitazone promoted adipocyte browning and inhibited autophagy. Rapamycin, an autophagy inducer, reversed adipocyte browning induced by rosiglitazone. Autophagy inhibition by rosiglitazone does not prevent mitochondrial clearance, which was considered to promote adipose whitening. Instead, autophagy inhibition increased p62 nuclear translocation and stabilized the PPARγ–RXRα heterodimer, which is an essential transcription factor for adipocyte browning. We found that rosiglitazone activated NRF2 in mature adipocytes. Inhibition of NRF2 by ML385 reversed autophagy inhibition and the pro-browning effect of rosiglitazone.

**Conclusion:** Our study linked autophagy inhibition with rosiglitazone-promoted browning of adipocytes and provided a mechanistic insight into the pharmacological effects of rosiglitazone.

## 1 Introduction

Obesity has become a worldwide epidemic in recent decades (Wang et al., 2021; Chen et al., 2023). Development of obesity is caused by hypertrophy and hyperplasia of white adipocytes, which are a key component in lipid-storing adipose. Distinct from white adipocytes, beige/brown adipocytes dissipate energy as heat and are considered beneficial in combating obesity (Gao et al., 2018; Ying and Simmons, 2020). Interestingly, white adipocytes can be transdifferentiated into beige adipocytes upon cold stimulation or sympathetic nerve activation, which is termed browning. Adipocyte browning can be observed in human adults (Finlin et al., 2018), highlighting its potential value in combating obesity (Ro et al., 2019).

Rosiglitazone is a thiazolidinedione used as an antidiabetic drug (Lehmann et al., 1995). Notably, treatment with rosiglitazone in inguinal white adipose tissue (iWAT) induces browning of adipocytes and alleviates high-fat diet-induced obesity in mice (Petrovic et al., 2010). It has been proposed that rosiglitazone activates peroxisome proliferator-activated receptor-γ (PPARγ), which enhances the transcription of genes essential for adipocyte browning (Digby et al., 1998). Nevertheless, the molecular and cellular alternations underlying the pro-browning effects of rosiglitazone have not been fully understood (Fayyad et al., 2019).

Autophagy is an evolutionary conserved process that degrades cellular molecules and organelles through the autophagosome–lysosome pathway (Reggiori and Klionsky, 2002; Wang and Klionsky, 2003; Levine and Klionsky, 2004). Accumulating evidence suggests that autophagy plays a crucial role in promoting brown-to-white adipocyte transition. White adipose tissue in adipocyte-specific *Atg7* knockout mice had increased browning features (Singh et al., 2009). In addition, adipocyte-specific deletion of *Atg5* or *Atg12* prevents loss of beige adipocytes in the whitening process after withdrawal of β3 adrenergic receptor agonist (Altshuler-Keylin et al., 2016). On the contrary, overactivation of BECN1, a pro-autophagy protein, by F121A mutation in primary adipocytes accelerates the loss of beige characteristics (Wu et al., 2023). Moreover, autophagy-induced mitochondrial clearance (mitophagy) also accelerates whitening of beige adipocytes (Taylor and Gottlieb, 2017; Lu et al., 2018). Rosiglitazone inhibits autophagy in hepatic cell line LX2 and spinal cord injury neurons, implying that rosiglitazone might play a role in autophagy inhibition (Li et al., 2017; Yum et al., 2023). Therefore, we aimed to explore whether and how rosiglitazone inhibits autophagy to induce adipocyte browning.

## 2 Materials and methods

### 2.1 Animal

Eight-week-old male C57BL/6 mice were fed on a high-fat diet (HFD) for 8 weeks. Then, two groups with five to six mice in each group were treated with the solvent (containing 0.5% DMSO, 40% PEG 400, 50% Tween-80, and 54.5% saline) or rosiglitazone (2.5 mg/kg) for 2 weeks subcutaneously on both sides of inguinal white adipose tissue under isoflurane anesthesia. The mice were kept on a HFD during drug administration. At the end of rosiglitazone treatment, the mice were euthanized by CO_2_ asphyxiation, and iWATs were collected for analysis.

All the animal experiments were approved by and conducted in accordance with the ethical guidelines of the Zhejiang University Animal Experimentation Committee and were in complete compliance with the National Institutes of Health Guide for the Care and Use of Laboratory Animals.

### 2.2 Cell culture

Two-week-old male C57BL/6 mice were euthanized by CO_2_ asphyxiation, and iWATs were isolated on ice and then digested with collagenase type Ⅰ for an hour in a 37°C water bath. Cells were filtered through a 70-μm filter and centrifuged for 5 min at 500 g. The cell pellet was resuspended and cultured in DMEM/F12 medium containing 10% FBS. On reaching confluence, the culture medium was changed with the induction medium (DMEM/F12 containing 10% FBS, 5 μg/mL insulin, 0.5 mM 3-isobutyl-1-methylxanthine, 1 μM dexamethasone, and 125 μM indomethacin) for 2 days. Then, it was changed into the differentiation medium (DMEM/F12 containing 10% FBS and 5 μg/mL insulin) for 6 days. For experiments pertaining to differentiating of adipocytes, 10 μM rosiglitazone was added in the differentiation medium for the indicated group. For experiments pertaining to mature adipocytes, the differentiation medium was changed with DMEM/F12 containing 10% FBS and 30 μM oleic acid for 4 days. Rosiglitazone (10 μM), 5 nM rapamycin, 10 μM chloroquine, or 10 μM ML385 were added in the media for 4 days in the indicated groups.

### 2.3 Histological analysis

iWATs from four mice of each group were isolated and fixed in 4% paraformaldehyde for 24 h and then embedded in paraffin. Tissue sections (6 μm) were stained with hematoxylin and eosin (H&E). Cells of the cultured adipocytes were grown on the microscope cover glasses. After the indicated treatment, cells were fixed in 4% paraformaldehyde for 10 min and stained with Oil Red O for 10 min. The experiments were repeated independently in at least triplicate. Both H&E staining of the iWAT sections and Oil Red O staining of the cultured adipocytes were visualized under 20-fold magnification by a Virtual Slide System (OLYMPUS, VS120). The area of lipid droplets was analyzed using ImageJ with at least 500 lipid droplets in randomly selected fields.

### 2.4 Cytoplasmic and nuclear protein extraction

Cytoplasmic and nuclear proteins from primary cultured adipocytes were extracted following the instructions of the Cytosolic and Cytoplasmic Protein Extraction Kit (Abbkine, KTP3001). Cells were washed with cold PBS, harvested with a spatula, and then centrifuged at 500 g for 5 min at 4°C. Two hundred microliters of working CESA (1:100 with protease inhibitor, 1:500 with DTT) was added and vortexed vigorously for 15 s to completely resuspend the cell pellets. After keeping it on ice for 15 min, 10 uL of pre-cooled CESB was added, vortexed for 10–15 s, and left on ice for 2 min. After centrifuging at 16,000 g for 5 min at 4°C, the supernatant extracted is the cytoplasmic protein. After resuspending the precipitate with pre-cooled working NES (1:100 for protease inhibitor and 1:500 for DTT), it is allowed to stand on ice for 30 min and vortexed for 15 s every 10 min. Then, it is centrifuged at 16,000 g for 5 min at 4°C. The supernatant extracted is a nuclear protein, which is then subjected to immunoblotting.

### 2.5 Immunoblotting and immunoprecipitation

iWATs and primary cultured adipocytes were homogenized using RIPA buffer (50 mM Tris, 150 mM NaCl, 1% Triton X-100, 1% sodium deoxycholate, and 0.1% SDS; pH 7.4). The Nuclear and Cytoplasmic Protein Extract Kit (Abbkine, KTP3001) and Mitochondria Protein Extract Kit (Sangon Biotech, C500051) were used for isolation of nuclear and mitochondria protein, respectively. The protein extracts were separated by SDS-PAGE and transferred onto nitrocellulose membrane (PALL, 66485). After blocking with 5% skim milk powder (BioFroxx, 3250GR500) for 1 h, the membranes were incubated with the following primary antibodies overnight at 4°C: BNIP3L (Cell Signaling Technology, 12396S; 1:1000), GAPDH (ABclonal, A19056; 1:3000), FUNDC1 (ABclonal, A16318; 1:1000), Histone H3 (ABclonal, A17562; 1:1000), LC3B (ABclonal, A19665; 1:1000), NRF2 (ABclonal, A0674; 1:1000), PPARγ (Proteintech, 16643-1-AP; 1:1000), Parkin (ABclonal, A0968; 1:1000), RXRα (ABclonal, A19105; 1:1000), SQSTM1/p62 (ABclonal, A19700; 1:1000), total OXPHOS (Abcam, ab110413; 1:1000), UCP1 (ABclonal, A21979; 1:1000), α-tubulin (ABclonal, A6830; 1:1000), and β-actin (ABclonal, AC026; 1:50000). After incubation with the secondary antibodies [HRP Goat Anti-Rabbit IgG (ABclonal, AS014; 1:5000) and HRP Goat Anti-Mouse IgG (ABclonal, AS003; 1:5000)] for 1 h, the blots were detected by the ECL Enhanced Kit (ABclonal, RM00021).

For immunoprecipitation experiments, primary cultured adipocytes were lysed using weak RIPA buffer (50 mM Tris, 150 mM NaCl, 1% NP-40, and 0.25% sodium deoxycholate; pH 7.4). The indicated primary antibodies [PPARγ (Proteintech, 16643-1-AP; 5 μg/mL) or Rabbit IgG (Beyotime, A7016; 5 μg/mL)] and then protein extracts were bound with Protein A/G Magnetic Beads (MCE, HY-K0202) for 2 h at 4°C. The immunoprecipitates were eluted by 1 × loading buffer (ABclonal, RM00001) boiled for 5 min at 95°C, and subjected to immunoblotting.

### 2.6 Real-time PCR

Total RNA from iWATs and cultured adipocytes was extracted using TRIzol reagent (Vazyme, R411-01) and an RNA Extraction Kit (Accurate Biology, AG21017), respectively. After quantification of RNA concentration by NanoDrop One (Thermo Scientific), the RNA was reverse-transcribed into cDNA using the Evo M-MLV RT Mix Kit (Accurate Biology, AG11728). Relative RNA expression was analyzed by LightCycler 480 Instrument Ⅱ (Roche) using SYBR Green Premix (Accurate Biology, AG11701). The reaction parameters were set at 95°C for 30 s, 95°C for 5 s, 60°C for 30 s (45 cycles), 95°C for 15 s, 60°C for 15 s, 95°C continuous for melt curves, and held at 40°C. The primers used for the experiments are listed in Supplementary Table S1.

### 2.7 Confocal imaging

For imaging of live adipocytes, cells were grown on confocal dishes (NEST). Before photographing, cells were treated with 100 nM MitoTracker Red (Invitrogen, M7512) for 30 min and Hoechst 33258 (Abcam, ab228550; 1:500) for 10 min. Cells were observed by using a confocal microscope (Leica, TCS SP8). At least five randomly selected fields were analyzed for each group by ImageJ.

### 2.8 Statistical analysis

Statistics were analyzed using GraphPad Prism 9.3. All the data were shown in mean ± SEM. A two-tailed Student’s t-test or one-way ANOVA was used for single or multiple comparisons. The nonparametric test was used for non-normally distributed data. A value of *p* < 0.05 was considered significantly different.

## 3 Results

### 3.1 Rosiglitazone inhibits autophagy while promoting adipocyte browning

**FIGURE 1:**
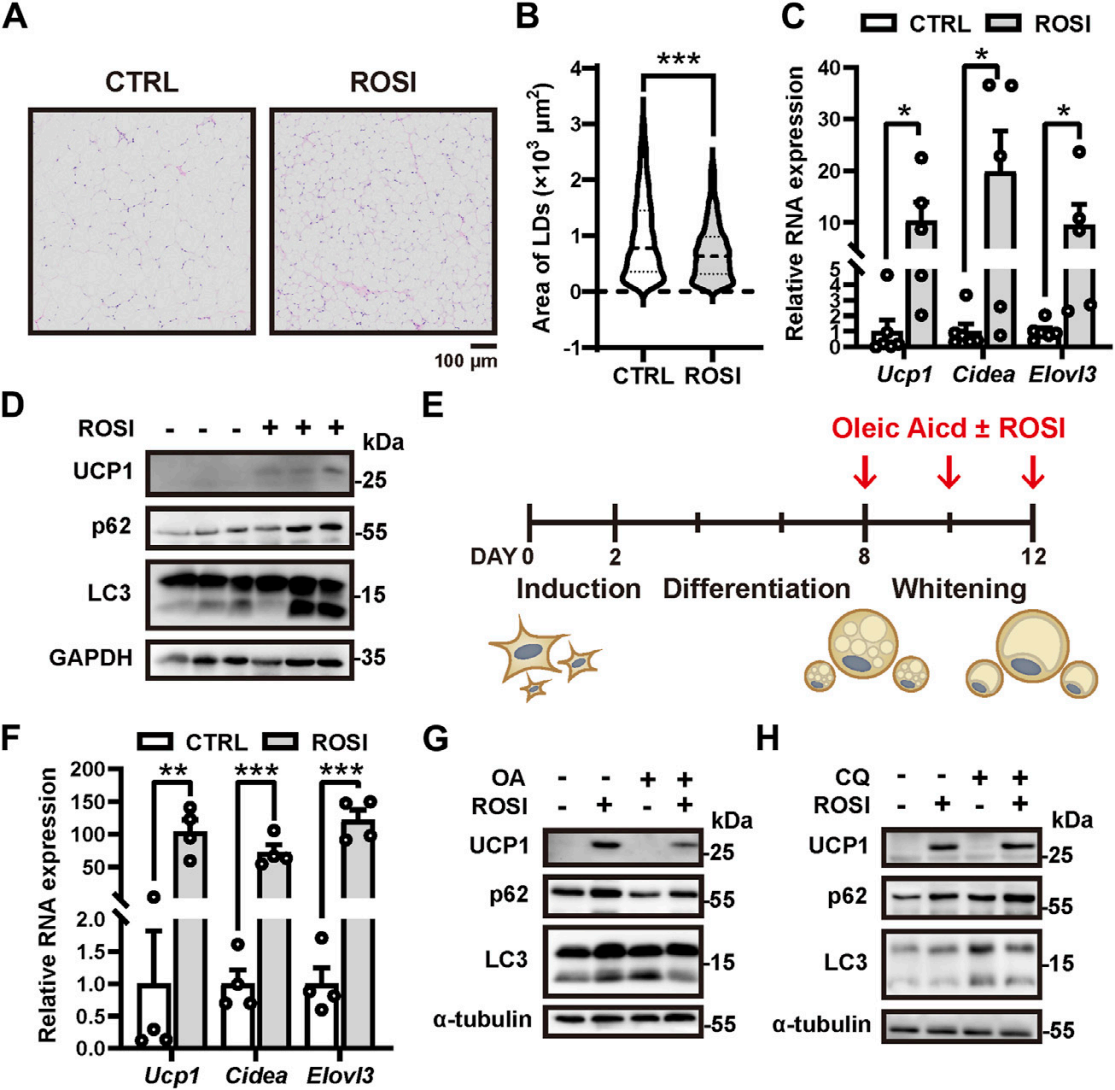
Rosiglitazone promotes white adipocyte browning while inhibiting autophagy in high-fat diet (HFD)-induced obesity mice and cultured adipocytes. **(A–D)** HFD-induced obesity mice were injected subcutaneously with 2.5 mg/kg solvent (CTRL) or rosiglitazone (ROSI) daily for 2 weeks. **(A)** Inguinal white adipose tissue (iWAT) depots were fixed and stained by H&E (*n* = 5–6 mice, per group). Scale bar, 100 μm. **(B)** Quantification of lipid droplet size in adipocytes from H&E staining shown in **(A)**. **(C)** Total RNA levels of *Ucp1*, *Cidea*, and *Elovl3* in iWAT were measured by qRT-PCR (*n* = 5–6 mice, per group). **(D)** UCP1, p62, and LC3 protein levels in iWAT were measured by Western blotting. GAPDH was blotted as a loading control. **(E)** Scheme for *in vitro* induction of adipocyte differentiation and whitening in **(F–H)**. When reaching confluence, stromal vascular fraction from iWAT was treated with the induction medium for 2 days and then switched to differentiation medium for 6 days. After differentiation, 30 μM oleic acid (OA) was treated for 4 days to induce mature adipocyte whitening. Any other reagents indicated in **(F–H)** were added together with OA. **(F)** Total RNA levels of *Ucp1*, *Cidea*, and *Elovl3* were measured by qRT-PCR in OA-treated cultured adipocytes from CTRL and ROSI (10 μM) groups. (*n* = 4 trials). **(G,H)** UCP1, p62, and LC3 protein levels were measured by Western blotting in matured adipocytes treated with OA, ROSI, and chloroquine (CQ, 10 μM) for 4 days. β-actin and α-tubulin were blotted as loading controls. Data in **(B,C, F)** are shown as mean ± SEM. Statistical analysis was performed by the Mann–Whitney test in **(B)** and *t*-test in **(C)** and **(F)**. *p < 0.05, **p < 0.01, and ***p < 0.001 *versus* CTRL.

Rosiglitazone (ROSI) promotes white adipocyte browning, but the molecular mechanism is not fully understood (Nedergaard and Cannon, 2014). High-fat diet (HFD)-treated mice were administrated rosiglitazone (2.5 mg/kg, *s.c.*, daily) for 2 weeks. We observed a significant reduction in the size of adipocyte lipid droplets (LD) in the inguinal white adipose tissue (iWAT), indicating white-to-beige adipocyte transition (Figures 1A, B). Moreover, transcription of browning-related genes *Ucp1*, *Cidea*, and *Elovl3* and the protein level of UCP1 were significantly enhanced in iWAT with ROSI treatment (Figures 1C, D). These results confirmed that ROSI induced adipose browning. To explore the involvement of autophagy activity in the pro-browning effects of ROSI, we detected the autophagy-related proteins in iWAT. It showed that both p62 and LC3-Ⅱ were accumulated with ROSI treatment, suggesting an autophagic flux blockage by ROSI in iWAT (Figure 1D). Moreover, primary cultured mouse adipocytes were incubated with oleic acid (OA) for 4 days to induce whitening, and ROSI was treated to adipocytes during D8 to D12 (Figure 1E). Consistent with the findings in mice iWAT, ROSI significantly upregulated the transcription of *Ucp1*, *Cidea*, and *Elovl3* and enhanced the protein level of UCP1 (Figures 1F, G). Furthermore, OA treatment alone decreased p62 and increased LC3-II, suggesting autophagy activation along with whitening, and these alternations were reversed by ROSI treatment (Figure 1G). To confirm the autophagic flux blockage by ROSI, cultured white adipocytes were simultaneously incubated with ROSI and lysosome inhibitor chloroquine (CQ). As expected, CQ alone induced dramatic accumulation of LC3-II and p62, which was observed to a less extent in the presence of ROSI, confirming the blockage of autophagy flux by ROSI in cultured adipocytes (Figure 1H). Taken together, these results suggested that rosiglitazone inhibits autophagy while promoting adipocyte browning.

### 3.2 Rosiglitazone induces adipocyte browning through autophagy inhibition

**FIGURE 2:**
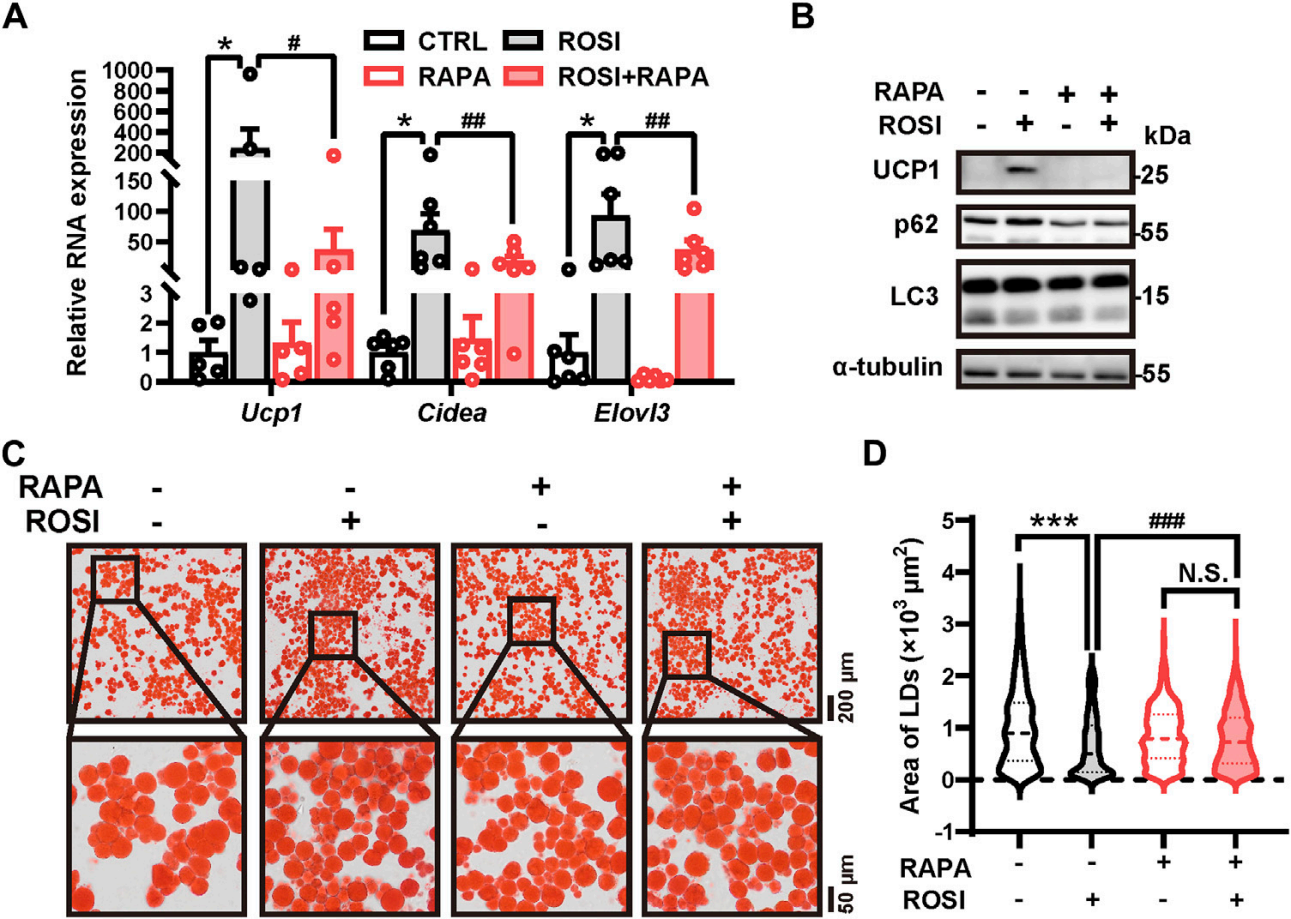
Rosiglitazone induces white adipocyte browning by inhibiting autophagy in oleic acid-treated adipocytes. **(A–D)** OA-induced whitened adipocytes were treated with rosiglitazone (ROSI, 10 μM) and rapamycin (RAPA, 5 nM) for 4 days. **(A)** Total RNA levels of *Ucp1*, *Cidea*, and *Elovl3* were measured by qRT-PCR (*n* = 5–6 trials). **(B)** UCP1, p62, and LC3 protein levels were measured by Western blotting. α-Tubulin was blotted as a loading control. **(C)** Lipid droplets in adipocytes were marked by Oil Red O staining (*n* = 3 trials). The square box shows the zoomed-in view of the indicated image. Upper scale bar, 200 μm; lower scale bar, 50 μm. **(D)** Quantification of lipid droplets’ size from Oil Red O staining shown in **(C)**. (*n* = 600–1,000 lipid droplets per group) Data in **(A)** and **(D)** are shown as mean ± SEM. Statistical analysis was performed by one-way ANOVA and nonparametric test with Dunn’s multiple comparisons. *p < 0.05, ***p < 0.001 *versus* CTRL. #*p* < 0.05, ##*p* < 0.01, and ###*p* < 0.001 *versus* ROSI.

To identify the contribution of autophagy inhibition in ROSI-induced browning of adipocytes, an autophagy activator rapamycin was employed. Rapamycin significantly abolished the effects of ROSI on browning-related gene expression, UCP1 protein level, and autophagy inhibition (Figures 2A, B). Additionally, as shown in Oil Red O staining, ROSI reduced the area of lipid droplets, indicating the transition to beige-like multilocular adipocytes, and pro-browning transition was also reversed by rapamycin (Figures 2C, D). These findings indicated that autophagy inhibition was required for the effects of ROSI in promoting adipocyte browning.

As autophagy inhibition may lead to deficits in adipogenesis, a process where adipocytes develop from precursor cells (Baerga et al., 2009; Zhang et al., 2009), we wonder whether ROSI inhibits autophagy and impairs adipogenesis. To this end, we applied ROSI to premature adipocytes for 8 days during their differentiation (Supplementary Figure S1A). It showed that more lipid droplets, organelles of mature adipocytes, were stained by Oil Red O in ROSI-treated adipocytes (Supplementary Figure S1B, C). In addition, ROSI did not suppress the expression of the late-stage adipogenesis marker FABP4 and even accelerated the appearance of UCP1 in differentiating adipocytes, indicating ROSI did not impair adipogenesis in differentiating adipocytes (Supplementary Figure S1D). Notably, neither ROSI alone nor in combination with rapamycin suppressed the transcription of browning markers, further suggesting that ROSI-inhibited autophagy has a limited impact on adipogenesis (Supplementary Figure S1E).

### 3.3 Rosiglitazone has no effect on mitophagy in differentiated adipocytes

**FIGURE 3:**
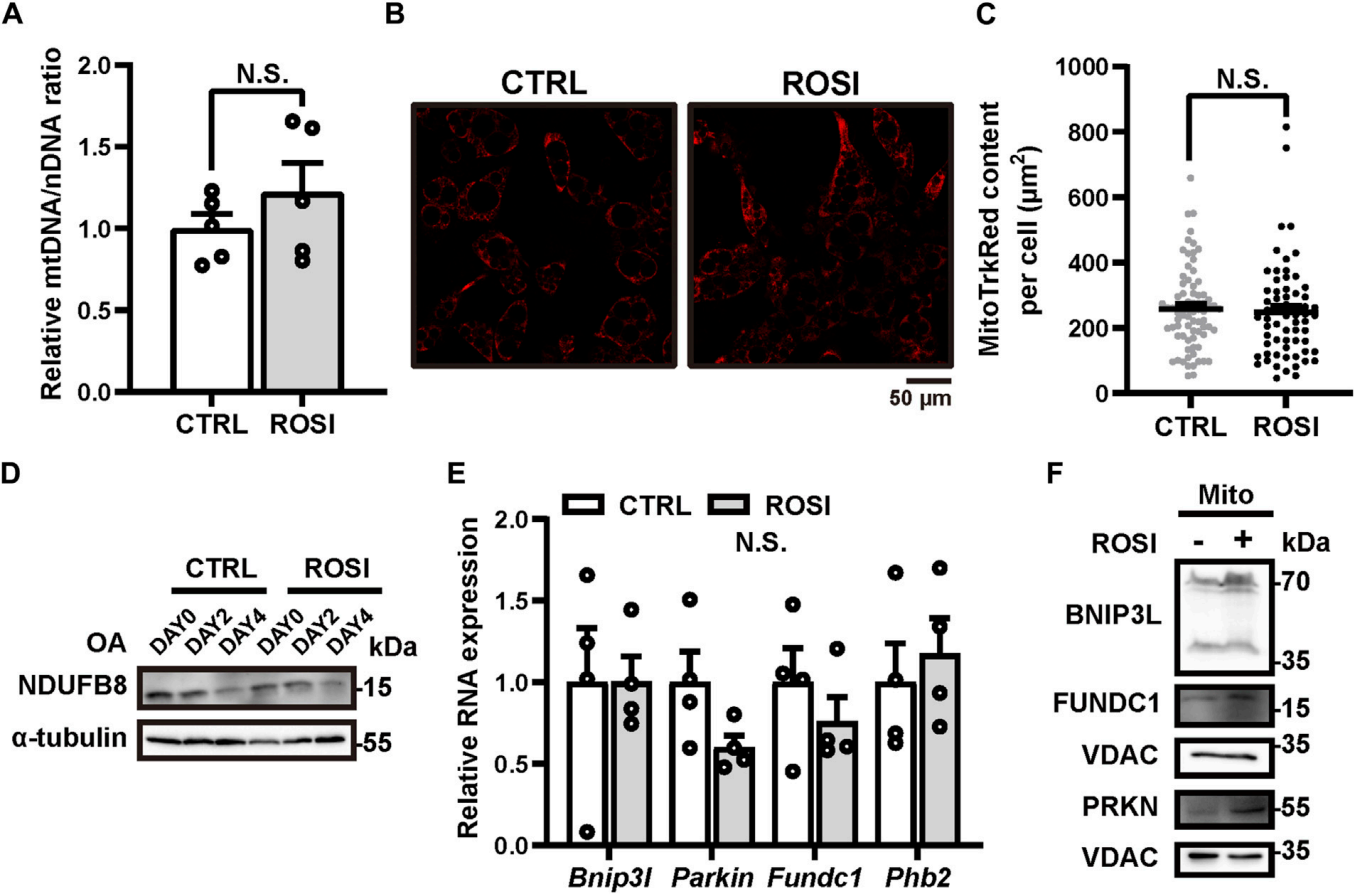
Rosiglitazone could not inhibit mitophagy in oleic acid-treated whitened adipocytes. **(A–F)** OA-induced whitened adipocytes were treated with rosiglitazone (ROSI, 10 μM) for 4 days. **(A)** Relative expressions of mitochondrial DNA (mtDNA) to nuclear DNA (nDNA) were measured by qRT-PCR (*n* = 5 trials). **(B)** Representative live image of MitoTracker Red staining in control or ROSI-treated adipocytes. Scale bar, 50 μm. **(C)** Quantification of MitoTracker Red fluorescence area showed in **(B)**. (*n* = 60–80 cells per group) **(D)** NDUFB8 protein levels were measured by Western blotting. α-Tubulin was blotted as a loading control. **(E)** Total RNA levels of *Bnip3l*, *Parkin*, *Fundc1*, and *Phb2* were measured by qRT-PCR (*n* = 4 trials). **(F)** BNIP3L, PRKN, and FUNDC1 protein levels in isolated mitochondria were measured by Western blotting. VDAC was blotted as a loading control. Data in **(A,C, E)** were shown as mean ± SEM. Statistical analysis by *t*-test shown in **(A)** and **(E)** and the Mann–Whitney test in **(C)**.

Mitochondria clearance via autophagy (mitophagy) promotes beige-to-white transition of adipocytes, and mitophagy inhibition maintains the brown-like phenotype of adipose (Altshuler-Keylin et al., 2016; Lu et al., 2018; Ro et al., 2019). We, therefore, hypothesized that ROSI inhibited mitophagy and, thus, promoted adipocyte browning. We measured the mitochondrial DNA (mtDNA) level to reflect the mitochondrial content in OA-treated adipocytes. Unexpectedly, ROSI did not alter mtDNA level (Figure 3A), and it did not change the quantity of MitoTracker Red-labeled mitochondria (Figures 3B, C), suggesting no mitophagy inhibition by ROSI in the adipocytes. Along with OA-induced adipocyte whitening, the protein level of mitochondria marker NDUFB8 gradually decreased, suggesting mitophagy activation. This trend of mitochondrial reduction was not affected by ROSI (Figure 3D). These results suggested that ROSI cannot reinforce mitophagy during OA-induced whitening of adipocytes. We next evaluated the RNA and protein levels of several mitophagy-related genes, *i.e., Bnip3L*, *Parkin*, *Fundc1*, and *Phb2* (Schweers et al., 2007; Geisler et al., 2010; Liu et al., 2012; Wei et al., 2017). It showed that ROSI neither downregulated the transcription nor the expression of these genes (Figures 3E, F), further suggesting that ROSI did not promote mitophagy during adipocyte whitening. Collectively, our findings indicated that mitophagy was not required for ROSI-induced adipocyte browning.

### 3.4 Rosiglitazone-mediated autophagy inhibition stabilizes the PPARγ-RXRα heterodimer

**FIGURE 4:**
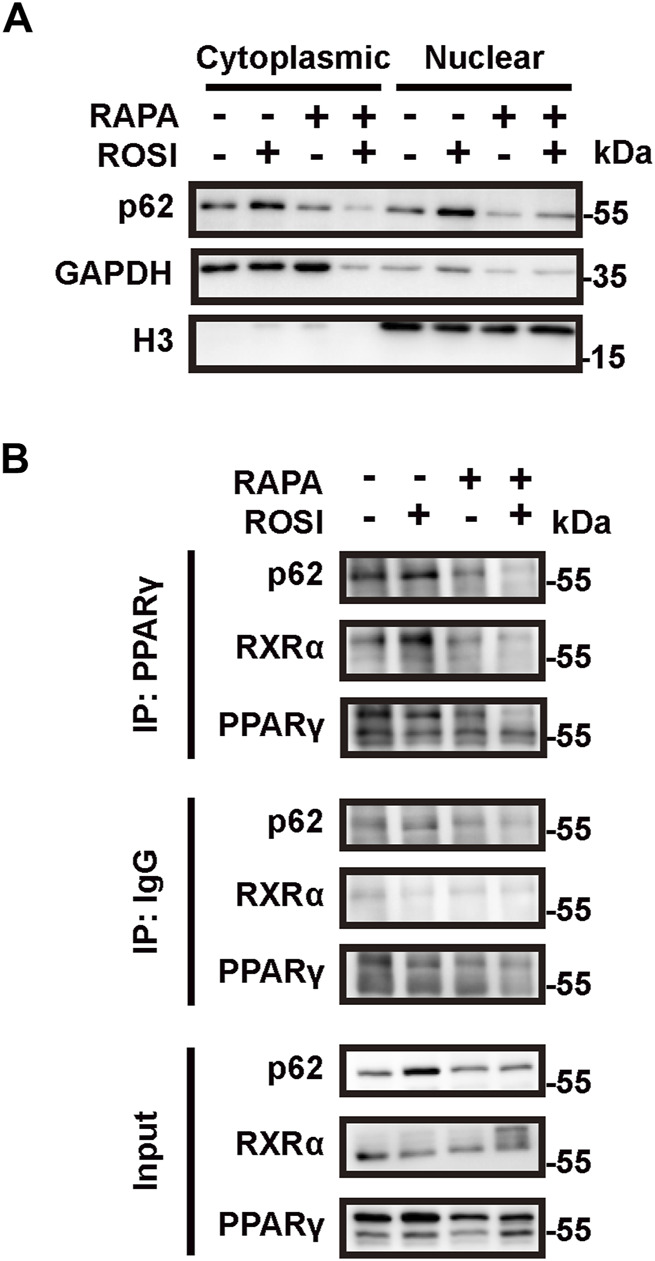
Rosiglitazone promotes nuclear translocation of p62 and stabilizes the PPARγ-RXRα heterodimer through autophagy inhibition. **(A,B)** OA-induced whitened adipocytes were treated with rosiglitazone (ROSI, 10 μM) and rapamycin (RAPA, 5 nM) for 4 days. **(A)** p62 protein levels in cytoplasmic and nuclear proteins of cultured adipocytes were measured by Western blotting. GAPDH was blotted as a loading control of the cytoplasmic protein, and H3 was blotted as a loading control of the nuclear protein. **(B)** Protein–protein interactions of p62, PPARγ, and RXRα in the nucleus were confirmed by immunoprecipitation.

We next explored through which mechanism ROSI-inhibited autophagy contributed to adipocyte browning. As an autophagy adapter protein, p62 is reported to fine-tune the transcriptional activity of the PPARγ-RXRα heterodimer and, thus, leads to expression of thermogenetic genes in brown adipocytes (Huang et al., 2021). To determine whether p62 was involved in ROSI-induced adipocyte browning, we isolated the nuclear protein of OA-treated adipocytes. We found p62 accumulation in the nucleus with ROSI treatment, which was reversed by rapamycin (Figure 4A). These data suggested that ROSI inhibited autophagy and led to p62 translocation to the nucleus. Moreover, the interactions between p62, PPARγ, and RXRα in the nucleus were also enhanced by ROSI and partly abolished by rapamycin (Figure 4B). These observations suggested that inhibition of autophagy by ROSI activated PPARγ-RXRα via promoting p62 nuclear translocation in adipocytes.

### 3.5 Activation of NRF2 by rosiglitazone inhibits autophagy and promotes adipocyte browning

**FIGURE 5:**
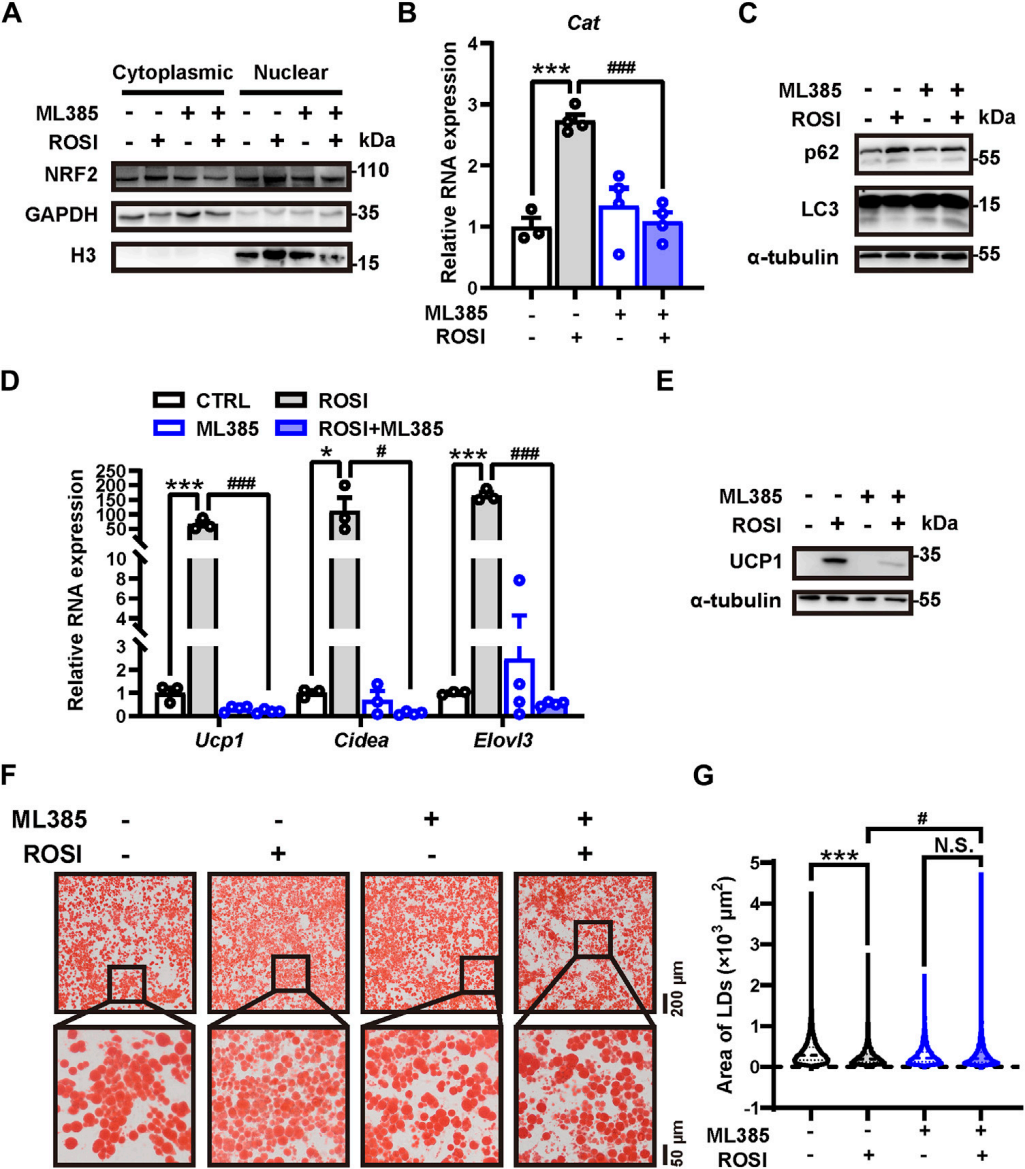
Rosiglitazone inhibits autophagy and promotes adipocyte browning through NRF2 activation **(A–G)** OA-induced whitened adipocytes were treated with rosiglitazone (ROSI, 10 μM) and ML385 (10 μM) for 4 days. **(A)** NRF2 protein levels in cytoplasmic and nuclear proteins of cultured adipocytes were measured by Western blotting. GAPDH was blotted as a loading control of cytoplasmic proteins, and H3 was blotted as a loading control of nuclear proteins. **(B)** Total RNA levels of *Cat* were measured by qRT-PCR (*n* = 3–4 trials). **(C)** p62 and LC3 protein levels were measured by Western blotting. α-Tubulin was blotted as a loading control. **(D)** Total RNA levels of *Ucp1*, *Cidea*, and *Elovl3* were measured by qRT-PCR (*n* = 3–4 trials). **(E)** UCP1 protein levels were measured by Western blotting. α-Tubulin was blotted as a loading control. **(F)** Lipid droplets in adipocytes were marked by Oil Red O staining (*n* = 3 trials). The square box shows the zoomed-in view of the indicated image. Upper scale bar, 200 μm; lower scale bar, 50 μm. **(G)** Quantification of lipid droplets’ size from Oil Red O staining shown in **(F)**. (*n* = 1,800–3,000 lipid droplets per group) Data in **(B,D, G)** are shown as mean ± SEM. Statistical analysis was performed by one-way ANOVA and nonparametric test with Tukey’s multiple comparisons in **(B)** and **(D)** and Dunn’s multiple comparisons in **(G)**. *p < 0.05; ***p < 0.001 *versus* CTRL. #*p* < 0.05; ###*p* < 0.001 *versus* ROSI.

**FIGURE 6:**
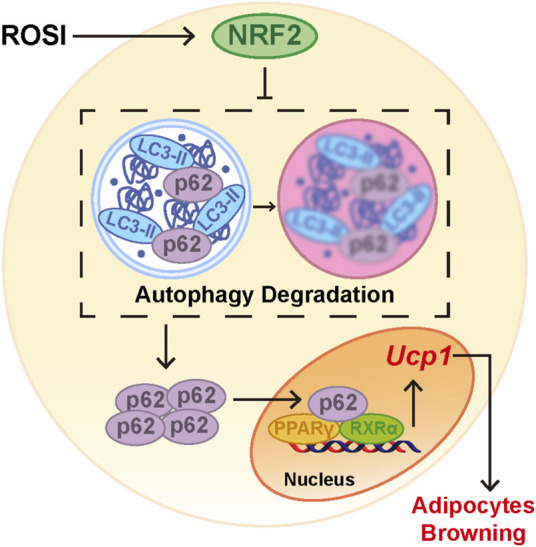
Rosiglitazone promoted adipocyte browning by NRF2-induced autophagy inhibition. When adipocytes were treated with rosiglitazone, nuclear translocation of NRF2 was activated. Activation of NRF2 inhibited autophagy and led to accumulation of p62 in the cytoplasm. Cumulative p62 translocated into the nucleus and bound with the PPARγ-RXRα heterodimer as a coactivator. Activated PPARγ increased the RNA expression of browning-related genes such as *Ucp1* and, thus, initiated adipocyte browning.

We next explored how ROSI inhibited autophagy in adipocytes. Nuclear factor erythroid 2-related factor 2 (NRF2) is a transcriptional factor that transcripts autophagy-related genes, including p62 (Liu et al., 2022). We found that ML385, an NRF2 inhibitor, prevented ROSI-induced p62 nuclear translocation (Figure 5A). The transcription of *Cat* mRNA, a target gene of NRF2, was upregulated by ROSI (Figure 5B), suggesting that ROSI activated NRF2. ML385 restored the inhibited autophagy flux in adipocytes with ROSI treatment (Figure 5C). Moreover, ROSI-induced browning characteristics including increase in RNA and protein levels of browning-related genes and the multilocular phenotype of adipocytes were also diminished by ML385 (Figures 5D–G). These data collectively indicated that ROSI inhibited autophagy through NRF2 activation and, thus, promoted adipocyte browning (Figure 6).

## 4 Discussion

As a PPARγ agonist, ROSI was reported to increase the expression of browning-related genes (Digby et al., 1998; Petrovic et al., 2010; Ohno et al., 2012), but the cellular and molecular mechanisms of how ROSI promotes adipocyte browning has not been fully elucidated. Autophagy has a vital role in beige-to-white adipocyte transition. Blockage of autophagy by deletion of autophagy-related genes or pharmacological methods induces adipocyte browning (Singh et al., 2009; Altshuler-Keylin et al., 2016). There are compounds that enhance adipocyte browning that were also reported to suppress autophagy, further highlighting the role of autophagy inhibition in promoting adipocyte browning (Leu et al., 2018; Duan et al., 2020). As ROSI leads to autophagy inhibition in hepatic cell line LX2 and spinal cord injury neurons (Li et al., 2017; Yum et al., 2023), we hypothesized whether ROSI promoted adipocyte browning through inhibition of autophagy. Our results showed that ROSI inhibited autophagy flux in high-fat diet-induced obese mice and oleic acid-induced whitened primary cultured adipocytes. Moreover, autophagy activation by rapamycin reversed the thermogenesis pattern and multilocular phenotype of white adipocytes under ROSI treatment, suggesting that autophagy inhibition is required in ROSI-induced adipocyte browning.

In addition to browning of adipocytes, autophagy inhibition could also lead to a deficiency in adipogenesis, the process through which adipocytes develop. *Atg5* or *Atg7*-deleted mouse embryonic fibroblasts are arrested at the early stage of adipogenesis (Baerga et al., 2009; Zhang et al., 2009). Autophagy is required to stabilize PPARγ2 by suppressing its proteasomal degradation and, thus, induce adipogenesis (Zhang et al., 2013). Interestingly, we found that ROSI inhibited autophagy without arresting adipogenesis. It has been documented that ROSI stimulates adipogenesis of mice and human preadipocytes (Lehmann et al., 1995; Yamauchi et al., 2001; Hutley et al., 2003). Another PPARγ agonist troglitazone could induce adipogenesis in *Ulk1* or *Atg5*-knockdown 3T3-L1 cells (Ro et al., 2013). Therefore, it seems that PPARγ activation by ROSI might have overwhelming effects on adipogenesis, regardless of whether autophagy is inhibited. This assumption should be verified by further investigation. Taken together, we exclude the possibility that ROSI-induced small multilocular phenotype of white adipocytes was due to autophagy inhibition-mediated adipogenesis deficiency.

Mitophagy has been reported to promote the beige-to-white adipocyte transition. Adipocyte-specific deletion of *Atg5* or *Atg12* rescued the mitochondria clearance process after β3 receptor agonist withdrawal and maintained beige adipocyte characteristics (Altshuler-Keylin et al., 2016). Similarly, mitophagy receptor *Parkin*-deficient mice showed reduced mitochondrial degradation and retained the beige adipocyte phenotype (Lu et al., 2018). These studies indicated that mitophagy inhibition facilitates adipocyte browning. However, our results suggested that ROSI has no impact on mitophagy. This result implied that mitophagy activity is dispensable for the pro-browning effects of ROSI. Consistently, ectopic PRKN expression failed to fully abolish ROSI-induced UCP1 in differentiated 3T3-L1 adipocytes (Taylor and Gottlieb, 2017). Nevertheless, knockdown of *Bnip3*, a mitophagy receptor, canceled ROSI-induced UCP1 expression in differentiated 3T3-L1 adipocytes (Choi et al., 2016). In addition to the non-mitophagy roles of PRKN and BNIP3, these controversial results may be attributable to the different measurements in revealing adipocyte transition. For example, the reduction of UCP1 in adipocytes, which indicates whitening, may result from its degradation within the mitochondria, where it is primarily located.

Canonical transactivation by PPARγ requires binding with RXRα as a heterodimer (Tontonoz et al., 1994), which interacts with coactivators upon agonist binding (Hernandez-Quiles et al., 2021). Autophagy adapter p62 serves as a coactivator of the PPARγ-RXRα heterodimer (Huang et al., 2021). Our results showed that ROSI-mediated autophagy inhibition triggered nuclear translocation of p62 and enhanced its binding with PPARγ and RXRα, suggesting that inhibiting autophagy can enhance the browning of adipocytes by increasing the expression and interaction of PPARγ coactivators. Similarly, PRDM16 was also stabilized by ROSI through inhibition of the ubiquitin–proteasome pathway, thus inducing a white-to-brown adipocyte transition (Ohno et al., 2012). In combination with the present study, these findings suggest that ROSI promotes adipocyte browning through fine-tuning of the interaction between PPARγ and its coactivators.

NRF2 was reported to play a vital role in adipocyte browning. Knockdown of *Nrf2* significantly reversed β3 receptor agonist CL316,243-induced adipose browning (Chang et al., 2021; Bauzá-Thorbrügge et al., 2023). Moreover, NRF2 activator tert-butylhydroquinone (tBHQ) and antioxidant N-acetylcysteine (NAC) induce adipocyte browning (Chang et al., 2021; Bauzá-Thorbrügge et al., 2023). However, the cellular and molecular mechanisms of how NRF2 activates adipocyte browning have not been fully understood. Our study suggested that autophagy inhibition is required for NRF2-induced browning, at least in ROSI-treated adipocytes. These findings are in line with the finding that ROSI inhibited autophagy and activated NRF2 in status epilepticus rats (Peng et al., 2021). However, another study indicated that autophagy impairment by *Atg3* knockout led to NRF2 activation in the adipose tissue (Cai et al., 2018), implying a positive feedback regulation of NRF2 by autophagy inhibition. p62 may serve as a hub in integrating autophagy and NRF2 signaling in adipocyte browning. As a substrate of autophagy, p62 accumulates in autophagy-impaired cells and competitively binds to Keap1, thus stabilizing NRF2 (Komatsu et al., 2010). NRF2 translocated in the nucleus could also increase transcription of p62 (Frias et al., 2020; Zhang et al., 2021). NRF2 or autophagy-related gene knockout mice may increase the reliability of the autophagy inhibitory effect of ROSI in future studies. Additionally, the transcriptional factor MiT/TFE was reported to enhance autophagy flux and promote browning of adipocytes (Altshuler-Keylin et al., 2016). It remains elusive whether MiT/TFE underscores the pro-browning effects of ROSI.

ROSI was used as an antidiabetic drug with the effects of promoting adipocyte browning and alleviating high-fat diet-induced obesity (Lehmann et al., 1995; Petrovic et al., 2010). It was reported that subcutaneous ROSI administration reduced iWAT size and promoted adipose browning in a diet-induced obesity mouse model. In combination with the present study, these investigations highlighted a promising role of ROSI in obesity treatment.

## 5 Conclusion

Our study revealed that ROSI promoted adipocyte browning through inhibition of autophagy. Autophagy inhibition by ROSI increased p62 nuclear translocation and stabilized the PPARγ-RXRα heterodimer for transcription of browning genes. We proposed that NRF2 activation was involved in autophagy inhibition by ROSI in adipocytes. The present study provided a new insight into the pharmacological mechanisms underlying ROSI-induced adipocyte browning.

## Data Availability

The original contributions presented in the study are included in the article/Supplementary Material; further inquiries can be directed to the corresponding author.
