# Supplementary material for: The autophagic regulation of rosiglitazone-promoted adipocyte browning
Source: Front Pharmacol. 2024 Jun 4;15:1412520. doi: 10.3389/fphar.2024.1412520 (PMC11184087; doi:10.3389/fphar.2024.1412520)
Supplement: Supplementary file 1 [file DataSheet1.docx]

Supplementary Material

# Supplementary Figures and Tables

## Supplementary Figures


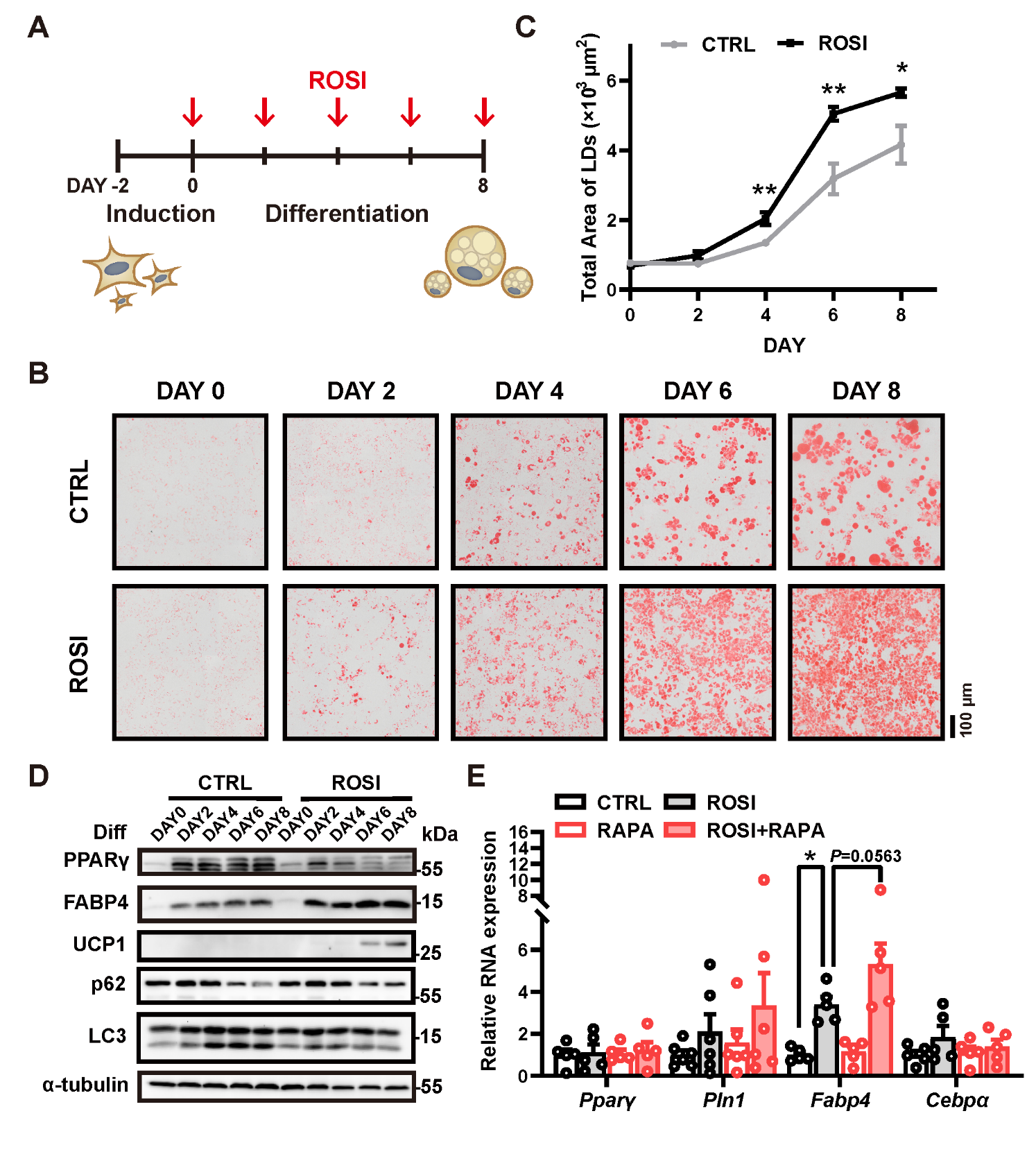


**Supplementary Figure 1.** **Rosiglitazone-mediated autophagy inhibition did not attenuate adipogenesis in both differentiating and mature adipocytes.**

**(A)** Scheme for in vitro induction of adipocytes differentiation and drug administration in **(B-D)**. When reached confluent, stromal vascular fraction from inguinal white adipose tissues was treated with induction medium for 2 days, then switched to differentiation medium for 8 days. Rosiglitazone (ROSI, 10 μM) was given in indicated group together with differentiation medium. **(B)** Lipid droplets from adipocytes were showed by Oil Red O staining at indicated time points (*n*=3 trials). Scale bar, 100 μm. **(C)** Total area of lipid droplets in every 10000 μm^2^ Oil Red O staining image showed in **(B)** was measured at indicated time points. (*n*=6 images per time point per group) **(D)** PPARγ, FABP4, UCP1, p62, LC3 protein levels were measured by western blotting at indicated time points. β-actin and α-tubulin were blotted as loading control. **(E)** Total RNA levels of *Pparγ*, *Fabp4*, *Pln1* and *Cebpα* were measured by qRT-PCR in OA-treated cultured adipocytes from indicated groups (*n*=4-5 trials). Rosiglitazone (10 μM) and rapamycin (5 nM) were incubated for 4 days. Data in **(C)** and **(E)** were shown as Mean ± SEM. Statistical analysis was performed by *t*-test in **(C)** and One-Way ANOVA and nonparametric test with Dunn’s multiple comparisons in **(E)**. **P*<0.05, ***P*<0.01 *versus* CTRL.

## Supplementary Tables

**Supplementary Table 1. Primer sequences used in real-time PCR**

| Gene | Species | Primer Sequences |
| --- | --- | --- |
| *Bnip3l* | mouse | Forward: ATGTCTCACTTAGTCGAGCCG |
|  |  | Reverse: CTCATGCTGTGCATCCAGGA |
| *Cat* | mouse | Forward: AGCGACCAGATGAAGCAGTG |
|  |  | Reverse: TCCGCTCTCTGTCAAAGTGTG |
| *Cebpα* | mouse | Forward: CAAGAACAGCAACGAGTACCG |
|  |  | Reverse: GTCACTGGTCAACTCCAGCAC |
| *Cidea* | mouse | Forward: TGACATTCATGGGATTGCAGAC |
|  |  | Reverse: GGCCAGTTGTGATGACTAAGAC |
| *Elovl3* | mouse | Forward: TTCTCACGCGGGTTAAAAATGG |
|  |  | Reverse: TCTCGAAGTCATAGGGTTGCAT |
| *Fabp4* | mouse | Forward: AAGGTGAAGAGCATCATAACCCT |
|  |  | Reverse: TCACGCCTTTCATAACACATTCC |
| *Fundc1* | mouse | Forward: CCCCCTCCCCAAGACTATGAA |
|  |  | Reverse: CCACCCATTACAATCTGAGTAGC |
| mtDNA | mouse | Forward: CCTATCACCCTTGCCATCAT |
|  |  | Reverse: GAGGCTGTTGCTTGTGTGAC |
| *Parkin* | mouse | Forward: TCTTCCAGTGTAACCACCGTC |
|  |  | Reverse: GGCAGGGAGTAGCCAAGTT |
| *Pecam* | mouse | Forward: ATGGAAAGCCTGCCATCATG |
|  |  | Reverse: TCCTTGTTGTTCAGCATCAC |
| *Phb2* | mouse | Forward: ATCCGTGTTCACCGTGGAAG |
|  |  | Reverse: CCCGAATGTCATAGATGATGGG |
| *Pln1* | mouse | Forward: GGGACCTGTGAGTGCTTCC |
|  |  | Reverse: GTATTGAAGAGCCGGGATCTTTT |
| *Pparγ* | mouse | Forward: TCGCTGATGCACTGCCTATG |
|  |  | Reverse: GAGAGGTCCACAGAGCTGATT |
| *Ucp1* | mouse | Forward: AGGCTTCCAGTACCATTAGGT |
|  |  | Reverse: CTGAGTGAGGCAAAGCTGATTT |
| *β-actin* | mouse | Forward: GGCTGTATTCCCCTCCATCG |
|  |  | Reverse: CCAGTTGGTAACAATGCCATGT |
